# Collectanea Medica

**Published:** 1812-04

**Authors:** 


### COLLECTANEA MEDICA,

CONSISTING OF

ANECDOTES, FACTS, EXTRACTS, ILLUSTRATIONS, QUERIES, SUGGESTIONS, &c.

RELATING TO THE

*History or the Art of Medicine, and the Auxiliary Sciences.*

---

### *An Essay upon Cinchonin, and its Influence upon the Virtue of Peruvian Bark, and other Barks. By* BERNARDINUS ANTHONY GOMES.

I. *History of the Knowledge hitherto obtained of Cinchonin.*— Cinchonin is a new vegetable principle, discovered for the first time in the bark of the officinal species of the genus cinchona, from which it derives its name. We are in some measure indebted to Dr. Maton for its discovery, as he was the first who remarked that the solutions of Peruvian bark gave a precipitate with tannin. At a latter period, Mr. Seguin, having found out the chemical character of tannin, which consists in giving a peculiar precipitate with gelatine, inferred, from the experiment of Dr. Maton, that there was gelatin contained in Peruvian bark; but he was deceived in this inference, as Dr. Duncan, jun. clearly pointed out. (Nicholson's Journal, vol. vi. p. 225.) And, in fact, a very easy experiment clearly proves that the precipitate of the solutions of Peruvian bark, produced by tannin, is owing to a principle not only different from gelatin, but from all others hitherto known.

Take some tincture of Peruvian bark, mix with it a sufficient quantity of water, and after a little time strain; to the strained liquor add the infusion of galls, a precipitate will be produced, which is totally soluble in alcohol.

This experiment shows that the principle of Peruvian bark, which is precipitated by infusion of galls or tannin, differs very much from gelatin and starch; for the precipitate which these two principles give with that re-agent are insoluble in alcohol.

It

It would appear that Dr. Duncan, though he made the remarkable discovery of cinchonin, never obtained it completely separated from the other principles of Peruvian bark, as he not only does not indicate the manner of obtaining it pure, either in his letter to Mr. Nicholson, in which he gives an account of his discovery, in the Annals of Medicine for the years 1803-4, wherein he wrote upon the same subject, or in the third edition of the Edinburgh New Dispensatory; but, moreover, when in this last work he mentions the properties of cinchonin, he says that it is "not acrid, soluble in alcohol and in water, precipitated by infusion of galls—precipitate soluble in alcohol." Now the first of these properties, the not being acrid, being negative, shews he did not obtain it pure; otherwise, by tasting it, we should have, instead of this negative property, a positive one. For the same reason he attributes to it solubility in water, which, it seems to me, it does not possess but by the agency of another principle.

The political state of Europe has not permitted me to see the memoir in which M. Vauquelin gives the account of his experiments upon the different kinds of cinchona or barks; but, judging by the extract which is inserted in the Medical and Surgical Review, vol. xv. p. 12, I am led to think this very dextrous and celebrated chemist never was able to obtain cinchonin free from other substances; for he ascribes to it properties which, according to my experiments, do not belong to it. "The principle which precipitates the infusion of oak bark and of galls," says M. Vauquelin (ibid, p. 12,) "is of a brown color, bitter, and less soluble in water than in alcohol, and precipitates emetic tartar, but not glue." In the subsequent pages it will be shewn, that these properties do not belong to pure cinchonin, but to its union with other vegetable principles.

It is not without some diffidence, that I venture to differ from Dr. Duncan, and M. Vauquelin, in what concerns their labours and opinions. However, I shall state, with candor and frankness, what I have found and judged different from what they thought.

But, before going further, I must own, that if, in what I am about to detail, I have at all advanced our knowledge of cinchonin, it is owing to the concise account of this principle which Dr. Duncan gave, and, as I mentioned above, principally to that of its chemical character consisting in giving, with the infusion of galls, a precipitate soluble in alcohol; which chemical character was certainly my chief guide in the investigation of its properties.

II. *Of the manner of extracting Cinchonin.*—Having remarked marked that the tincture of cinchona, when not previously precipitated by water, gives, on being mixed with infusion of galls, a precipitate which is not completely dissolved by alcohol; and, having observed, moreover, that the precipitate of the tincture of cinchona by water is dissolved by potassa, I concluded that this precipitate was not resin, and that it might be extractive, which has the property of losing its solubility in proportion as it becomes more oxygenated, and that of being soluble in water by means of potassa, even when it is oxygenated. Having remarked also, that potassa formed, in the aqueous solution of the alcoholic extract of cinchona, a white precipitate, which was redissolved by alcohol, and showed other properties of cinchonin, I concluded that it might be obtained by oxygenating and rendering insoluble the greatest part of the extractive of the cinchona, which accompanies it in the tincture, or alcoholic solution, separating by means of water what was or becomes insoluble, then oxygenating the residuum, and separating it afterwards from the cinchonin by potassa. I do not pretend to defend the accuracy of this theory, I intend only to indicate what suggested to me the following process to obtain cinchonin.

Take tincture of Peruvian bark; let it evaporate to the consistency of an extract; mix with it, by means of a glass rod, various and successive portions of distilled water, and strain them successively until the water pass almost colorless and insipid. Evaporate the whole of the filtered liquors till the extract be obtained; add to this, successive portions of a well saturated solution of potassa, and strain successively through the same filter until the lixivium pass colorless, or the residuum become white; wash the residuum in the same filter with a small portion of cold water, and let it dry.

By this process there remains on the filter a substance which is white when in a state of greater purity, and pale or reddish when less so. When white it is powdery, and is easily detached from the filter. It is also bitter, inflammable, very little soluble in water, but soluble enough when recently prepared, in sulphuric ether, alcohol, in diluted sulphuric, nitric, and muriatic, acids, in the acetic, oxalic, citric, *malic*, but not in the *tartaric*, acid*. From these solutions, which are made without effervescence, it is precipitated by the infusion of galls, and the precipitate is white and capable of being redissolved by alcohol. This substance is of course the cinchonin of Dr. Duncan, but, even

* I doubt of its solubility in malic acid, and of its insolubility in tartaric acid, for I suspect the purity of the acids I employed.

when white, it is not sufficiently pure, for it remains always more or less contaminated with coloring matter, with the small threads of the filter, and, even after being washed, with some potassa.

III. *Process to purify Cinchonin.*—Impure cinchonin, (§ II.) is dissolved in the best alcohol, then it is strained, and to this solution an equal quantity of distilled water is added; the mixture is left in a glass loosely covered with paper, until the odor of alcohol be not perceived; it is then strained, and the residuum left to dry on the filter, forming very fine, small, and white, filiform crystals.

IV. *Varieties observed in this Purification.*—The result of the preceding process varies somewhat according to the quality of the bark from which the cinchonin has been extracted. When the alcoholic solution (§ III.) is of the cinchonin of the red bark, or other Peruvian barks, not of the thick kinds, on the addition of the water there results a transparent mixture; and it is only after standing some time that the filiform crystals begin to float in the liquor, and to precipitate and increase in number and size as the alcohol evaporates; and at the same time tuberculous incrustations are formed on the sides of the vessel of a crystalline appearance when humid, but opaque, more or less colored, and without brilliancy, when dry.

If the alcoholic solution (§ III.) is of the cinchonin of the thick barks *(calissayas)* of Lima and of Santa Fé, immediately upon the addition of the water the liquor becomes opaque and milky, and a resinous reddish substance, in form of drops of oil, but concrete, is observed to swim on the surface of the liquor instead of crystals, and the sides of the vessel are covered with incrustations, as in the preceding case.

In fine, when the alcoholic solution (§ III.) is of the cinchonin of the bark called *huanuco*, which is distinguished more or less by tubercles or protuberances on the external surface, not only there appear many crystals of a silver brightness, but the incrustations are in a great manner composed of the same crystals, and very white and silver-like.

V. *Examination of the filiform Crystals.*—1. These very fine and minute filiform crystals, rubbed between the fingers, are converted into a white and very subtle powder, resinous to the touch, as if we were rubbing between the fingers powder of colophony.

2. They are insipid and inodorous, but they seem to dissolve in the saliva.

3. Exposed to the flame of a candle by means of a glass rod, they decrease in volume, exhale smoke with a peculiar odor, odor, not disagreeable, melt, taking a chesnut color, and burn with a clear and white flame.

4. They are insoluble in water either cold or warm, for water agitated with them and strained, does not give any precipitate with the infusion of galls.

5. Mixed with the cold aqueous infusion of *cinchona pubescens* of Brazil (which does not give any precipitate with the infusion of galls, but becomes turbid with the solution of glue, and dark greenish-brown with the solution of sulphate of iron), it forms a turbid and gelatinous liquor, which, when strained, gives, with the infusion of galls, a precipitate redissoluble by alcohol.

6. They are soluble in alcohol, sulphuric ether; in the diluted sulphuric, nitric, and muriatic, acids; in the acetic, oxalic, citric; in the gallic (§ V. 5.); in the malic; not in the tartaric.

7. The acid solutions give a white precipitate with the infusion of galls, which is completely redissolved by alcohol. If to the same solutions we add any of the three alkalies, a precipitate is formed in white flakes, which is redissolved by alcohol.

8. The solution in sulphuric acid is speedy, complete, and without effervescence. The precipitate, which the solution of potassa forms in this, is as white as lime, insipid, inflammable like the crystals, is dissolved slowly, but completely, in alcohol, from which it is precipitated by water in smaller crystals, but very similar to the primitive ones.

9. Lime-water does not seem to precipitate the solution of the crystals in muriatic acid, even though it be added in excess. This mixture gives, with the infusion of galls, a precipitate which is only dissolved in part by alcohol.

10. From these properties it seems to me, that the following conclusions may be deduced: 1st, That the crystals are a pure vegetable principle. This appears from the regular and crystalline form which they assume, from their complete solubility in sulphuric acid, and from the precipitate which potassa forms in this solution, having the same properties as the crystals, (§ V. 8.) 2d, That this pure principle is the cinchonin of Dr. Duncan, since it gives, with the infusion of galls, a white precipitate capable of being dissolved by alcohol, (§ V. 7.) 3d, That this principle, in its insolubility in water, its inflammability and solubility in alcohol and ether, bears some analogy to resin, but differs from it by its crystallisation and solubility in the acids, (§ V. 6.) 4th, That by these last properties it is somewhat analogous to camphor, from which it differs, however, in being without odor (§ V. 2.), in being precipitated crystal-

 lised lised from the alcoholic solution (§ III. and V. 8.); in having greater specific gravity, since it sinks in water (§ IV.); in giving a precipitate with the infusion of galls, &c. 5th, That, by having singular and peculiar properties, it is, as Dr. Duncan thought, a vegetable principle different from all others hitherto known.

VI. *Examination of the Incrustations.*—1. These are in form of small protuberances, white while humid; opaque, commonly red, and without any brilliancy, when dry; they seem resinous when they are masticated, and of a pungent and bitter flavor.

2. Exposed to the flame of a candle they melt, and burn better than the crystals, with a clear and white flame.

3. They are sparingly soluble in cold water, for when agitated with them and strained, it gives a white precipitate with the infusion of galls.

4. They are not totally dissolved in the mineral and vegetable acids, in which the crystals are completely soluble (§ V. 6.), and they are also precipitated from these solutions by the infusion of galls.

5. A solution in diluted sulphuric acid, after being strained, is transparent, and gives a white precipitate with potassa, but not so clear as that of the crystals.

6. This precipitate (§ V.) is bitter, and burns with the same odor and residuum as the filiform crystals; it is not completely dissolved by alcohol, leaving a reddish residuum. This alcoholic solution does not give a precipitate with eight times its quantity of water, and thus diluted it gives a precipitate with the infusion of galls, which is re-dissolved by alcohol; it does not give a precipitate with the solution of emetic tartar, and does not change the color of paper tinged with turnsol, or with the flowers of mallows.

7. From these properties, it seems to me, that it may be inferred, 1st, That these incrustations contain cinchonin, or the substance of the filiform crystals, (§ V.) for they give, with the infusion of galls, a precipitate which is re-dissolved by alcohol (§ VI. 4 and 6). 2d, That they differ from the crystals in being in the form of protuberances, and in having color and taste (§ VI. 1), in burning better (§ VI. 2), in being sparingly soluble in water (ib. 3), in being not completely soluble in the acids (ib. 4), in giving a less clear precipitate (ib. 5), bitter, not completely soluble in alcohol, and the residuum being of a reddish color (ib. § VI. 6). Lastly, in this alcoholic solution not being precipitated by eight times its quantity of water (ib.). It contains, besides cinchonin, a substance which imparts to it color, flavor, and solubility in water, which is not very soluble in alcohol, or in the acids,

 in in which, however, it is sparingly dissolved, and is precipitated with the cinchonin, in fine, which impedes its crystallisation, leaving to it scarcely a mark of this property, when in the form of small protuberances. 3d, That the incrustations have neither a free acid nor alkaline base (§ VI. 6). 4th, That cinchonin, at least in this combination, does not precipitate the solution of emetic tartar (ib.).

VII. *Of the liquor which remains after the Crystallisation and Incrustation.*—This liquor, being strained and left to evaporate spontaneously, in the course of some days assumes a gelatinous appearance, but it never assumes the form of a jelly; it has a bitter taste, and at last emits the smell of orange and cinnamon-flowers.

This liquor changes paper tinged with flowers of mallow to a green color; it gives, with the infusion of galls, the same precipitate with the crystals and incrustations, and it effervesces with sulphuric acid.

These phenomena show, that this liquor contains carbonate of potassa, cinchonin, and the principle which communicates bitterness to the incrustations, and makes them sparingly soluble in water.

Whence does the pleasant smell of orange and cinnamon-flowers proceed, which is only perceived after a long evaporation?

VIII. *Of the Combination in which Cinchonin is found in various Vegetables.*—Cinchonin is not a principle peculiar to cinchona, as its name indicates, which therefore is not well appropriated. According to Dr. Duncan it is also found in angustura, columba, ipecacuanha, black-pepper, Guinea-pepper, and in opium. I found it in the red bark; in that of *huanuco;* in the coarse barks of Lima and Santa Fé; in a kind of bark brought from Brazils, which is similar to, if not identical with, that of Lima; in the barks of *Portlandia hexandria;* in a light, thin, and smooth, bark (of which I shall speak in another publication relating to cinchonas), which was brought from the province of *Goiazes,* with the improper name of cinchona, and which seems to me identical with another bark given to me in Brazil, and which I was told was brought from *Minas Geraes,* and was there known under the name of bark of the land orange-tree; lastly, in a coarse bark, red within, and heavy, which I shall describe at the same time with Goiazes bark, which was brought from the village of *Camamu* through Bahia, with the name, equally improper, of cinchona. But I did not find cinchonin in two kinds of cinchona discovered in the province of Rio de Janeiro, and which are, according to Dr. Vicent Gomes, of that city, and our celebrated botanist, Dr.

Dr. Brotero, the barks of the *cinchona pubescens* and of the *cinchona macrocarpa.* This circumstance farther shows the impropriety of the name *cinchonin*, which it is yet convenient to preserve, in order to avoid confusion.

Of the barks above-mentioned, all those which I examined and which contained cinchonin, yielded this principle to water as well as to alcohol and wine. Hence perhaps Dr. Duncan and M. Vauquelin concluded that it was soluble in water and in alcohol; but, being certainly, as I have said (§ V. 4.), when pure, by itself insoluble in water, the conclusion is, that it exists in those barks combined with another principle which makes it soluble in water, as we observe in § VI. 3. 6. There are many reasons to suspect this solvent to be an acid in the true cinchonas; for, besides cinchonin being soluble in various acids, and of there being in all the cinchonas marks of acidity, it is by potassa that it is precipitated in the process of extraction (§ II.). It is even probable that this acid is the gallic; for, on examining the precipitate with potassa, a substance is found which gives a brown color to the solution of sulphate of iron.

But, if the gallic acid be the solvent of the cinchonin in the cinchonas, it is not so in all the other barks which contain it; for the aqueous solutions of the alcoholic extracts of the barks of Goiazes and of Camamu, not only do not redden the tincture of turnsol, but on the contrary they make it green, and the cinchonin of these barks, and of the Portlandia hexandria, is not precipitated by potassa as that of the cinchonas (§ II.).

Besides this difference in the solvent of cinchonin, I believe there is yet another. The solvent of cinchonin in the cinchonas loses by oxygenation, or by the action of the air, more and more of its solubility, not only in water, but also in alcohol. This observation leads me to think, that it partakes a great deal of, or bears a great analogy to, the extractive of M. Fourcroy. It does not seem then improbable that it may be in different states of oxygenation in different barks, and that by being perhaps more oxygenated in the coarse ones or *calissayas*, and by being found in some of them mixed with resin, it happens, that, in the purification of the cinchonin of these barks, a milky precipitate is formed without obtaining crystals (§ IV.). It may also happen, that the solvent of the cinchonin in the barks of Portlandia, Goiazes, and Camamu, is less oxygenated, or less capable of oxygenation, than that of the cinchonas. This conjecture becomes probable, on observing that the tinctures of the barks of Goiazes and Portlandia scarcely become turbid with water; and that of Camamu bark, though becoming so, gives a less precipitate precipitate than the tincture of cinchona. On the other hand, the potassa which precipitates the cinchonin in the recent aqueous extracts of the alcoholic extracts of the cinchonas, does not give any precipitate with the recent aqueous extracts of the alcoholic extracts of Camamu bark, but it gives some with the old extracts of this bark, and none at all with the extracts, either recent or old, of the barks of Goiazes and Portlandia.

But we shall quit this subject for the present, till new experiments enable us to proceed from probability to chemical demonstration. In the mean time I beg leave to reason, from the data I now possess, though they are not all those which are requisite, upon an important problem in medical practice.

IX. *Of the Influence of Cinchonin upon the Virtues of Vegetables.*—It is well known that M. Seguin, supposing the cinchonin of cinchona to be gelatin, considered that the febrifuge principle, as he called it, was the supposed gelatin of this bark; and, in his illusion, he attempted to substitute animal gelatin in place of cinchona. Not having seen the memoir of M. Seguin on the febrifuge principle of cinchona, but only an extract from it (Medical and Physical Journal, Vol. XI. p. 215.), I do not know the facts he had to form and support his opinion, but I believe that it is not altogether destitute of foundation; for, when I was attending the military and naval hospitals, I received orders from the offices of the respective secretaries of state to try different barks, unknown in European medicine, which had been brought from Brazils under the name of cinchona. At that time I had not yet tried any chemical experiment with respect to cinchonin, and it was quite unknown to me whether the barks of Brazil contained it or not. Thus, without any previous knowledge, I tried successively these barks in fevers, particularly in those of the intermittent type, and I found that the barks of Camamu, Goiazes, Portlandia hexandria, one kind of cinchona of the Brazils, and different cinchonas of the Spanish America, were remarkably febrifuge. At the same time, I remarked with regret and astonishment, that two true species of cinchona, brought also from Brazils, possessed nothing, or almost nothing, of that quality.

Reflecting upon this result of clinical observations, it occurred to me that a comparative chemical analysis might explain that remarkable difference, and even indicate the principle which renders the good Peruvian bark eminently febrifuge, it appearing to me very probable, that all the barks which were remarkably antifebrile, might have a principle ciple or common circumstance, which ought to be entirely or almost wanting in the barks which were not febrifuge.

To observe how far the truth of this conjecture could be extended, I began to try chemical experiments upon all the Spanish cinchonas, and the three cinchonas and other three barks of Brazils above mentioned; and I found that all the Spanish cinchonas of our shops, one of those of the Brazils, which I had observed was febrifuge, and the barks of Goiazes, Camamu, and Portlandia, contained cinchonin, and that this principle was not to be found in the other two cinchonas from Rio Janeiro, that is, in the barks of the *cinchona macrocarpa*, and of the *cinchona pubescens*, which possessed very little or nothing of the febrifuge quality.

Hence, from all these cinchonas, and also three other barks, which contain cinchonin, being febrifuge, and from two true species of cinchona, which do not contain it, having very little or no pretensions to that title, I am inclined to conclude that cinchonin is the principle which renders cinchona, and the other vegetable substances containing it, eminently febrifuge.

This conclusion, however, seems to be contradicted by what M. Vauquelin says (Med. and Chir. Review, Vol. XV. p. xiii.), "As the property of precipitating tannin is not common to all the cinchonas, they therefore do not exclusively derive from that their febrifuge power, for there are many which do not precipitate tannin, and yet we know that they cure fevers."

This quotation seems to indicate that the cinchonas which contain cinchonin, and those which do not contain it, are equally febrifuge; but the case is so far from being so, according to M. Vauquelin himself, that he says, in another place, "It even seems, that the principle which precipitates the infusion of oak-bark and galls is febrifuge; for it is in general well known in medicine, that those species of cinchona which produce this effect are the best."

Thus the observations of the illustrious French chemist, far from contradicting, confirm my conclusion with respect to cinchonin, to which I ascribe only the febrifuge preeminence of cinchona, and not exclusively the febrifuge power; for it is universally known, that, before the discovery of cinchona, and even after it, fevers were cured with bitters and compositions without cinchona. But, from general practical observation, every person reckons these febrifuges so inferior to good cinchona, that, in dangerous intermittents, they have recourse to it in preference to any other.

But, as cinchonin is insipid, inodorous, and always found in vegetables in combination with other principles, which render render it bitter, soluble in water, &c. it becomes a question whether it be febrifuge by itself, or only an essential part of the febrifuge principle.

If it is a well-known fact, as Dr. Duncan affirms, that angustura, which, according to him, contains cinchonin, does not cure intermittent fevers, (New Dispensatory, p. 157,) we ought to conclude, that cinchonin is merely an essential part of the febrifuge principle of vegetables. This conclusion derives great probability from the practical observation, that, in general, cinchona given in powder acts more powerfully than any of the preparations of this bark, and from the medicinal qualities of ipecacuanha, opium, &c. which contain also cinchonin (§ VIII.), being different.

But what is merely probable is not demonstrated, and in matters of such importance probabilities do not suffice; evidence is necessary. It is then of importance to determine, by decisive clinical experiments, what is the combination, either natural or artificial, that renders cinchonin eminently febrifuge, or whether, in spite of its insipidity and insolubility in water, it be febrifuge by itself, as is possibly the case; for the tartrate of potassa and antimony, and the antimonial powders, &c. are insipid, and yet stimulate the stomach in a remarkable manner. Besides, although it is insoluble in water, it does not seem to be so in saliva, and in the acids of the stomach, &c.

I shall, in the mean time, observe, with regard to this, that the infusion of some of the cinchonas, which contain cinchonin, precipitates the solution of glue, and turns blackish the solution of sulphate of iron; that the infusion of the red cinchona, and that of the bark of Goiazes, produce with this last re-agent the same effect, but do not precipitate the solution of glue; lastly, that the infusion of the bark of Camamu, neither changes the color of the solution of sulphate of iron, nor does it precipitate glue. From this I must infer, that the extract or solvent of the cinchonin, in some of the cinchonas, contains gallic acid and tannin; that the red bark and that of Goiazes contain gallic acid but no tannin; that of the bark of Camamu contains neither gallic acid nor tannin; and, as all these barks are excellent febrifuges (§ IX. 2.), it follows that the febrifuge principle, or essential part of the febrifuge principle of vegetables, neither exists in the gallic acid, which is to be found in all of them except in that of Camamu, nor in the tannin which exists in some of the cinchonas in very small quantity, as was shewn by Dr. Maton, and the celebrated Davy, and which does not exist at all in the red cinchona, or in the barks of Goiazes and of Camamu; consequently, Mr. Wilkinson, M. Fourcroy, and and Dr. Westring of Sweden, have not sufficient reason to ascribe to tannin the prerogative of being febrifuge.

Hence it is only the cinchonin which can now be recognised as the principle that renders cinchona eminently febrifuge; and, as it is not contained in all the species of cinchona, it is of advantage, in choosing this drug, to attend not only to the sensible qualities, but also to examine if it contains cinchonin, which is very easily done, either by the experiment of the first section, or by mixing the aqueous infusion of the cinchona, which is to be examined with another infusion of galls. If the result of this mixture is a white precipitate, there is cinchonin, and so much the more abundant as the precipitate is more speedy or more copious.—*Edin. Jour.*

---

*Extracts from Holinshed's Chronicles, Reign of Edward VI.*

A remarkable interview is recorded by Holinshed, under the reign of Edward VI., between Bishop Ridley and the young Monarch, which ended in the establishment of the Royal Hospitals.—The condition of *the poor* was very wretched in the interval between the dissolution of the monasteries and the passing of the celebrated statute commonly called the 39th of Elizabeth. The measures taken with them during the reign of her brother Edward were very severe. They were liable to be *branded with a hot iron,* and to be reduced to *actual slavery.* Could we not, if necessary, recite the very words of the terrible act of parliament, inflicting such punishment on beggars, we should hardly have ventured to mention what must now seem altogether incredible. Such a law was very soon repealed. The first dawnings of a due distinction amongst paupers, will be found in the admirable classification stated below, the joint work of the venerable Bishop Ridley, the Lord Mayor of London at that time (Sir Richard Dobbes), and certain Aldermen and Commons of the city.—It should seem that this arrangement was not made without consideration; for, although King Edward did not live above two days after completing the plan, Dobbes was mayor in 1551, but the king died July 6, 1553. Many persons of high character appear in the list of mayors about that period: the Greshams, Sir Martin Bowes, Sir Rowland Hill, Sir Thomas White, and others. The whole extract is very interesting on many occasions.

" It chanced the reuerend father in God maister Doctor Ridleie, then Bishop of London, to preach before the king's maiestie at Westminster. In the which sermon he made a pitieful and godlie exhortation to the rich, to be merciful vnto the poore, and also to mooue such as were in authoritie, to trauell by some charitable waie waie and meane, to comfort and releeue them. Wherevpon the king's maiestie being a prince of such towardnesse and vertue for his yeares, as England before neuer brought forth, and the same also being so well reteined and brought vp in all godlie knowledge, as well by his deere uncle the late protector, as also by his vertuous and learned scholemaisters, was so carefull of the good gouernment of the realme, and chieflie to doo and prefer such things as most speciallie touched the honor of almightie God. And, vnderstanding that a great number of poore people did swarme in this realme, and chieflie in the citie of London, and that no good order was taken for them, did suddenlie and of himselfe send to the said bishop as soon as his sermon was ended, willing him not to depart vntill that he had spoken with him, (and this that I now write was the verie report of the said bishop Ridleie), who according to the king's commandment gave his attendance. And, so soone as the king's maiestie was at leasure, he called for him, and made him to come vnto him in a great gallerie at Westminster, where there was present no more persons than they two, and therefore made him sit downe in one chaire, and he himselfe in another, (as it seemed) were before the comming of the bishop there purposelie set, and caused the bishop (maugre his teeth) to be couered, and then entered communication with him in this sort.

" First giuing him most hartie thanks for his sermon and good exhortation, he therein rehearsed such speciall things as he had noted, and that so manie, that the bishop said: 'Trulie, trulie (for that was commonlie his oth), I could never haue thought that excellencie to haue beene in his grace, that I beheld and saw in him.' At the last, the king's maiestie much commended him for his exhortation for the reliefe of the poore. 'But, my lord, (saith he) ye willed such as are in authoritie to be carefull thereof, and to deuise some good order for their reliefe, wherein I thinke you meane me; for I am in the highest place, and therefore am the first that must answer unto God for my negligence, if I should not be carefull therein; knowing it to be the expresse commandement of almighty God, to haue compassion of his poore and needie members, for whome we must make an accompt vnto him. And trulie, my lord, I am before all things most willing to trauell that waie, and I doubt nothing of your long and approued wisedom and learning, who hauing such good zeale as wisheth helpe vnto them, but that also you haue had some conference with others what waies are best to be taken therein, the which I am desirous to understand, and therefore I praie you saie your mind.'

" The bishop thinking least of that matter, and being amazed to heare the wisdome and earnest zeale of the king, was (as he said himselfe) so astonied that he could not well tell what to saie: but, after some pause, said that, as he thought at this present for some entrance to be had, it were good to practise with the citie of London; because the number of the poore there are verie great, and the citizens are manie and also wise; and he doubted not but they were also both pityfull and mercifull, as the maior and his brethren and other the worshipfull of the said citie. And that, if it would please the king's maiestie to direct his gratious letter unto the maior of London, willing him to call unto him such assistants as he should thinke meet, to consult of this matter, for some order to be taken therein, he doubted not but good should follow thereof. And he himselfe promised the king to be one himself that should earnestlie trauell therein.

" The king forthwith not onelie granted his letter, but made the bishop tarie untill the same was written, and his hand and signet set thereunto, and commanded the bishop not onelie to deliver the said letter himselfe, but also to signifie unto the maior that it was the king's speciall request and expresse commandment, that the maior should therein trauell, and, as soon as he might conveniently, give him knowledge how farre he had proceeded therein. The bishop was so ioious of the hauing of this letter, and that he had now an occasion to trauell in that good matter, wherein he was marvellous zealous, that nothing could more have pleased and delighted him: wherefore the same night he came to the maior of London, who then was sir Richard Dobs, knight, and delivered the king's letter, and shewed his message with effect.

" The maior not onelie ioiouslie receiued this letter, but with all speed agreed to set forward this matter, for he also fauoured it verie much. And the next daie, being Mondaie, he desired the bishop of London to dine with him: and against that time, the maior promised that he would send for such men as he thought meetest to talk of this matter, and so he did. And sent first for two aldermen and six commoners, and afterward were appointed more, to the number of foure and twentie. And in the end after sundrie meetings (for by meane of the good diligence of the bishop it was well followed) they agreed upon a booke that they had deuised, wherein first they considered of nine special kinds and sorts of poore people, and those same brought in these three degrees.

| | |
|---|---|
| *Three degrees of Poore:* - - | The poore by impotencie,<br>Poore by casualtie,<br>Thriftless poore. |

| | |
|---|---|
| 1. The poore by impotencie are also divided into three kinds, that is to saie - - - - | 1. The fatherlesse poore man's child,<br>2. The aged, blind, and lame,<br>3. The diseased person by leprosie, dropsie, &c. |
| 2. The poore by casualtie are of three kinds, that is to saie | 4. The wounded souldier,<br>5. The decaied housholder,<br>6. The visited with greeuous disease. |
| 3. The thriftless poore are three kinds in like wise, that is to saie - - - - - - - - - - - - | 7. The rioter that consumeth all,<br>8. The vagabond that will abide in no place,<br>9. The idle person, as the strumpet and others. |

" For these sorts of poore were prouided three seuerall houses. First, for the innocent and fatherlesse, which is the beggers child, and and is indeed the seed and breeder of beggerie, they prouided the house that was late Graie-friers in London, and is now called Christes hospitall, where the poore children are trained in the knowledge of God, and some vertuous exercise, to the ouerthrow of beggerie. For the second degree, is prouided the hospitall of saint Thomas in Southworke, and Saint Bartholomew in West Smithfield, where are continuallie at least two hundred diseased persons, which are not onelie there lodged and cured, but also fed and nourished. For the third degree they prouided Bridewell, where the vagabond and idle strumpet is chastised and compelled to labour, to the ouerthrow of the vicious life of idleness. They prouided also for the honest decaied housholder, that he should be relieued at home at his house, and in the parish where he duelled, by a weeklie releife and pension. And in like manner they prouided for the lazer to keepe him out of the citie from clapping of dishes, and ringing of bels, to the great trouble of the citizens, and also to the dangerous infection of manie, that they should be relieved at home at their houses with seuerall pensions. Now after this good order taken, and the citizens by such meanes as were deuised, willing to further the same, the report thereof was made vnto the king's maiestie: and his grace for the aduancement hereof, was not onelie willing to grant such as should be the ouerseers and gouernours of the said house, a corporation and authoritie for the gouernment thereof: but also required that he might be accounted as the chiefe founder and patrone thereof, and, for the furtherance of the said worke, and continuall maintenance of the same, he of his meere mercie and goodness granted, that were before certeine lands given to the maintenance of house of the Sauoie founded by king Henrie the seuenth, for the lodging of pilgrims and strangers, and that the same was now made but a lodging of loiterers, vagabounds, and strumpets, that laie all daie in the fields, and at night were harboured there, the which was rather the maintenance of beggarie, than the reliefe of the poore, gaue the same lands, being first surrendred into his hands by the maister and fellowes there (which lands were of the yearelie value of six hundred pounds) vnto the citie of London, for the maintenance of the foundation aforesaid.

"And for a further reliefe, a petition being made to the king's maiestie for a licence to take in mortmaine, or otherwise, without license, lands to a certaine yearlie value, and a space left in the patent for his grace to put in what summe it would please him, he, looking on the void place, called for pen and inke, and with his owne hand wrote this summe, in these words (four thousand marks by yeare) and then said in the hearing of his councell: 'Lord God, I yeeld thee most hartie thanks, that thou hast given mee life thus long, to finish this worke to the glorie of thy name.' After which foundation established, he lived not above two daies, whose life would have been wished equall to the patriarchs, if it might haue pleased God so to have protracted the same."

*Importance*

### *Importance of the Stamina and Pistilla in the Production and Ripening the Seed of Plants.*

In the middle of the last century, in a hot-house at Berlin, there was a date palm, which blossomed very luxuriantly every year with flowers containing pistilla only, but never produced any seed or fruit that came to perfection. In a hot-house at Leipsic there was one of the same species that produced blossoms with stamina only. In 1749, a branch of this tree in flower was sent from Leipsic to Berlin, and suspended over the palm tree there, that produced only pistilla, and that year, for the first time, it bore perfect fruit and seed; some of which were sent to Linnæus in Sweden, who raised other trees from them at Upsal.

The importance of these two parts of a flower to the maturing the fruit of the palm was known to the ancients, and the trees, bearing flowers with stamina only, were carefully planted among those which bore the pistilla, that the dates might come to perfection; and among the moderns, where this fruit is an article of food, it is now no less scrupulously attended to. M. Michaux, in his travels in Persia, has observed, that in the contentions and civil commotions in that country for the dominion of empire, the different parties which were alternately victorious, in order more speedily to reduce the inhabitants of the provinces, burned all the palm-trees that produced stamina; and famine would have been the consequence had not the Persians previously taken the precaution to preserve a great quantity of the pollen for the purpose of fructifying the fruit-bearing trees which produce only pistilla. It also appears from the same author, that the pollen which had been preserved for this purpose, had been kept for eighteen years without losing its fertilising property.

To illustrate the importance of the stamina and pistilla, Linnæus made a decisive experiment on the *Glaucium phœnicium* (red horned poppy). From this plant he stripped off the stamina from two separate flowers, and afterwards carried the pollen of a third to one of those which he had deprived of its stamina. The seed of that poppy came to maturity which he had so fertilised, and the other did not: the experiment was frequently repeated with the same result.

---

### *Remarkable Attachment to Bees in an Idiot Boy.*

About the year 1755, there was an idiot boy in the village of Selborne in Dorsetshire, who from a child shewed a strong propensity to bees; they were his food, his amusement, his sole object, and, as people of this cast have seldom more than one point in view, so this lad exerted all his few faculties ties in this one pursuit. In the winter he dozed away his time within his father's house, by the fire side, in a kind of torpid state, seldom departing from the chimney-corner; but in the summer he was all alert, and in quest of his game in fields, and on sunny banks. Honey-bees, humble-bees, and wasps, were his prey wherever he found them: he had no apprehension from their stings, but would seize them *nudis manibus*, and at once disarm them of their weapons, and sack their bodies for the sake of their honey bags. Sometimes he would fill his bosom between his shirt and his skin with a number of these captives; and sometimes would confine them in bottles. He was a very *merops apiaster*, or bee-bird, for he would slide into the bee-gardens, and, sitting down before the stools, would rap with his finger on the hives, and so take the bees as they came out. He has been known to overturn hives for the sake of honey, of which he was passionately fond. Where metheglin was making, he would linger round the tubs and vessels, begging a draught of what he called bee-wine. As he ran about he used to make a humming noise with his lips, resembling the buzzing of bees. This lad was lean and sallow, and of a cadaverous complexion; and, except in his favorite pursuit, in which he was wonderfully adroit, discovered no manner of understanding. Had his capacity been better, and directed to the same object, he had perhaps abated much of our wonder at the feats of a more modern exhibitor of bees. He died before he arrived at manhood.

---

### *Tobacco and Snuff in New Spain.*

The manufacture of cegars and snuff in New Spain (a royal right,) annually amounts to more than 6,200,000 livres tournois (253,060l. sterling). The manufactures of Mexico and Queretaro are the most considerable. The following is an account of the whole manufacture during 1801 and 1802.

| *Tobacco manufactured in New Spain.* | In 1801. *Piastres.* | In 1802. *Piastres.* |
|---|---|---|
| Value of Tobacco manufactured agreeably to Sales . . . . | 7,825,913 | 7,686,834 |
| Expence of Manufacture . . | 1,299,411 | 1,285,199 |
| Salaries of Officers . . . | 798,452 | 794,586 |
| Price of Tobacco purchased from the Mexican Husbandmen . . | 626,319 | 594,229 |
| Net revenue *(liquido)* of the crown on the Sale of Tobacco . . | 3,993,834 | 4,092,629 |

On my passage through Queretaro, says Humboltz, I visited the great manufacture of cegars *(fabrica de puros y cigarros)*,

*cigarros)*, in which 3000 people, including 1900 women, are employed. The halls are very neat, but badly aired, very small, and consequently excessively warm. They consume daily in this manufacture 130 reams *(resmas)* of paper, and 2770 pounds of tobacco-leaf. In the course of the month of July 1803, there was manufactured to the amount of 185,288 piastres; viz. 2,654,820 small chests *(caxillas)* of cegars, which sell for 165,926 piastres, and 289,799 chest of *puros* or cegars which are not enveloped in paper. The expence of manufacturing in the month of July alone, amounted to 31,789 piastres. It appears that the royal manufacture of Queretaro annually produces more than 2,200,000 piastres, in *puros* and *cigarras*.

